# Assessing Kidney Injury Biomarkers and OTA Exposure in Urine of Lebanese Adolescents Amid Economic Crisis and Evolving Dietary Patterns

**DOI:** 10.3390/toxins17120577

**Published:** 2025-11-30

**Authors:** Rouaa Daou, Maha Hoteit, Jad Chémali, Nikolaos Tzenios, Nassim Fares, André El Khoury

**Affiliations:** 1 Centre d’Analyses et de Recherche (CAR), Unité de Recherche Technologies et Valorisation Agro-Alimentaire (UR-TVA), Faculty of Sciences, Campus of Sciences and Technologies, Saint Joseph University of Beirut, Beirut PO Box 17-5208, Lebanon; rouaa.daou1@usj.edu.lb; 2 PHENOL Research Program, Faculty of Public Health, Section 1, Lebanese University, Beirut P.O. Box 6573, Lebanon; m.hoteit@ul.edu.lb; 3 Department of Primary Care and Population Health, University of Nicosia Medical School, Nicosia P.O. Box 24005, Cyprus; 4 INSPECT-LB (Institut National de Santé Publique, d’Épidémiologie Clinique et de Toxicologie-Liban), Beirut 1103, Lebanon; 5 Laboratoire de Cancérologie et d’agents cancérogènes, Faculty of Medicine, Saint Joseph University of Beirut, Beirut P.O. Box 17-5208, Lebanon; jad.chemali@usj.edu.lb; 6 Faculty of Public Health, Charisma University, London EC1V 7QE, UK; 7 Laboratory of Research in Physiology and Pathophysiology, Faculty of Medicine, Saint Joseph University of Beirut, Beirut P.O. Box 17-5208, Lebanon; nassim.fares@usj.edu.lb

**Keywords:** mycotoxins, dietary exposure, ochratoxin A (OTA), Lebanese adolescents, public health risk, kidney injury biomarkers

## Abstract

Although ochratoxin A (OTA) contamination has been previously reported in Lebanon, this study is the first worldwide to assess its potential impact on renal health among adolescents aged 10 to 18 years. In this cross-sectional study, the aim was to evaluate the levels of OTA, OTα, and kidney injury biomarkers, as well as creatinuria and total proteinuria, while correlating these findings with dietary patterns. Urinary concentrations of OTA, its main metabolite ochratoxin α (OTa), the three renal injury biomarkers (N-acetyl-β-D-glucosaminidase [NAG], Kidney Injury Molecule-1 [KIM-1], and human lipocalin-2 [NGAL]), and two renal function indicators (creatinine and total protein) were quantified. Associations between biomarker levels and dietary intake patterns were also evaluated. OTA and OTα were detected in 14.2% and 59.5% of urine samples, respectively. NGAL and NAG were found in all participants at low concentrations, with the NAG-to-creatinine ratio exceeding the clinical threshold in 1.5% of samples, while KIM-1 was detected in 86% of participants. A weak positive correlation between urinary OTα and NAG suggests subtle renal alterations possibly linked to OTA exposure. Correlations between biomarker levels and food consumption were generally weak and positive. Estimated dietary intake (EDI) of OTA generated from consumption patterns was shown to be less than the probable dietary intake (PDI) calculated from urinary OTA. However, this presented a limitation, as EDI was calculated from previous contamination data in Lebanon. Overall, these findings indicate that while renal injury biomarkers were present at low levels, they may reflect early kidney stress not yet manifesting as overt pathology and highlight the need for strengthened regulatory measures to limit OTA contamination in foods available on the Lebanese market and for longitudinal studies to confirm these preliminary findings.

## 1. Introduction

Mycotoxins are secondary metabolites of filamentous fungi that can contaminate crops both in the field and during storage. Their presence in the global food system poses significant threats to food security, public health, and economies, particularly in warm and humid climates where fungal proliferation is prevalent [1]. These molecules are chemically stable, making them resistant to food processing methods and difficult to eliminate from contaminated commodities [2]. Exposure to mycotoxins can lead to acute or chronic health effects, with developing countries disproportionately affected due to limited mitigation strategies and reliance on subsistence farming [3]. Humans are exposed either directly through consumption of contaminated food or indirectly via animal-derived products such as meat, milk, and eggs [4].

Ochratoxin A (OTA) is one of the most concerning mycotoxins due to its widespread occurrence. Produced by *Aspergillus* and *Penicillium* species, OTA thrives under diverse environmental conditions and is commonly found in cereals, grains, dried fruits, coffee, beer, and cocoa [5]. Post-harvest contamination often occurs under improper storage conditions characterized by elevated temperatures and humidity levels [6]. OTA is nephrotoxic and has been implicated in Balkan Endemic Nephropathy (BEN), a chronic renal disease with strong familial and geographical clustering in endemic rural regions [7]. The International Agency for Research on Cancer (IARC) has classified OTA as a possible human carcinogen (Group 2B) due to its demonstrated carcinogenic effects in animal studies [8].

Upon ingestion, OTA exhibits prolonged systemic circulation due to its high plasma protein binding affinity (~99%), resulting in a half-life of approximately 35 days [9]. It undergoes metabolism, primarily in the intestines, liver, and kidneys, yielding ochratoxin alpha (OTα), a hydrolyzed derivative considered significantly less toxic. Given its nephrotoxic properties, OTA exposure can lead to kidney injury. Although specific biomarkers for OTA-induced renal damage have not been identified, general markers such as N-acetyl-β-D-glucosaminidase (NAG), Kidney Injury Molecule-1 (KIM-1), human lipocalin-2 (NGAL), creatinine (Cr), and total protein (TP) have been associated with renal damage [10]. Evaluating ratios, such as the urinary NAG-to-creatinine or total protein-to-creatinine ratios, provides a more accurate assessment of kidney injury severity [11,12]. Measuring OTA and its metabolite OTα alongside these biomarkers and their relative ratios in urine could offer valuable insights into exposure levels and renal effects.

Previous studies conducted in Lebanon have consistently confirmed the presence of OTA in food products, particularly cereals [13,14,15,16,17,18,19]. Amid Lebanon’s ongoing economic crisis-driven dietary shifts toward low-cost food, the population increasingly relies on affordable staples, heightening the risk of OTA exposure. Children and adolescents aged 10–19 are particularly vulnerable due to their dietary habits, developing physiology, and lower body weights [20]. In Lebanon, although contamination with OTA was previously documented, no biomonitoring data exists that links OTA exposure to early kidney injury. Thus, the aim of this study was to evaluate the levels of OTA, OTα, and kidney injury biomarkers N-acetyl-β-D-glucosaminidase (NAG), Kidney Injury Molecule-1 (KIM-1), human lipocalin-2 (NGAL), as well as creatininuria and total proteinuria, while correlating these findings with dietary patterns. To achieve this goal, we analyzed urine samples from 400 children and adolescents across different Lebanese regions and used a food frequency questionnaire (FFQ) to assess their dietary habits.

## 2. Results

### 2.1. Population Characteristics

Sociodemographic characteristics and health characteristics of school children and adolescents’ participants are shown in Table 1 and Table 2, respectively. The general characteristics of parents are shown in Table 3. Of the adolescent participants, 56% were females. The majority (92.5%) had both parents as their primary caregivers. Regarding working status, the majority (93.7%) reported not working. More than half of the participants had a normal body mass index (68.8%), while only 4.5% were underweight, and the rest were either overweight or obese. Concerning supplement intake, most participants reported not currently taking supplements of any of the vitamins and minerals examined. Significant differences between males and females existed for vitamin D (*p*-value = 0.004) and iron (*p*-value = 0.007) intake, with more females taking these supplements than males. Among school children and adolescents who reported taking iron supplements (n = 106), about one-third (n = 36, 34%) reported that they stopped taking the supplements for financial reasons. More than half of the households were not crowded (52%), and most parents reported not having a job (55%).

### 2.2. Food Groups Consumption and Comparison to Three Different Diets’ Recommendations

#### Food Groups Consumption

Food groups consumption of adolescent participants per gender and per age group are shown in Table 3 and Table 4, respectively. Overall, ‘bread, cereals, and grains’ was the most consumed food group (333.98 g/d), followed by fruits (253.62 g/d) then vegetables (236.64 g/d). Male participants had a significantly higher consumption of the ‘bread, cereals, and grains’ (*p*-value < 0.001), ‘dairy products’ (*p*-value = 0.004), ‘eggs’ (*p*-value < 0.001), ‘added sugars’ (*p*-value = 0.003), and ‘added fats and oils’ (*p*-value = 0.037) groups compared to female participants, while females had a significantly higher consumption of the ‘vegetables’ (*p*-value = 0.046) group. As for age categories, older adolescents (14–18 years) had a significantly higher consumption of the ‘vegetables’ (*p*-value = 0.031), ‘processed meat’ (*p*-value = 0.006), ‘fish’ (*p*-value = 0.012), ‘added sugars’ (*p*-value = 0.003), ‘hot beverages’ (*p*-value < 0.001), and ‘alcoholic beverages’ (*p*-value = 0.023) groups than younger adolescents (10–13 years).

### 2.3. Dietary Exposure Analysis

Table 5 shows the dietary intake of every food group with the mean OTA contamination reported in Lebanon in different studies. Accordingly, EDI was calculated in different food groups, and the total exposure to OTA from food was found to be 4.92 ng/kg bw/day. Mean OTA levels were obtained from studies reported in “Mycotoxins in Lebanese Food Basket: Database on Occurrence and Exposure” [21].

### 2.4. Exposure and Renal Data

#### 2.4.1. Mycotoxin Biomarkers

Table 6 presents the mean of the ochratoxin biomarkers, namely urinary OTA and OTα, that were found in 57 (14.2%) and 238 (59.5%) samples out of 400, respectively. The mean concentration in all samples of OTA and OTα was found to be 1.01 and 3.54 μg/L, respectively.

#### 2.4.2. Kidney Injury Biomarkers

Table 7 presents the average kidney biomarkers in urine, where Kim-1 was present at an average of 325.7 pg/mL, and NGAL and NAG at 57.4 pg/mL and 175.3 mU/L, respectively. Accordingly, the Kim-1 and NGAL-to-creatinine ratio did not exceed the cut-off in any of the samples, while only 6 individuals (1.5%) had a NAG-to-creatinine ratio higher than the cut-off value of 8.7 ng/mg Cr.

#### 2.4.3. Renal Function Indicators

Table 8 presents renal function indicators, where the average of total protein, creatinine, and TP/Cr ratio were equal to 11.6 mg/dL, 124.4 mg/dL, and 0.11, respectively. The TP/Cr ratio was specifically calculated, as many studies have shown a strong positive correlation between this method and the 24 h proteinuria level, making the TP-to-Cr ratio calculation a more practical and convenient method to assess kidney health [22]. According to the International Pediatric Nephrology Association (IPNA), the cut-off value of 0.2 mg TP/mg Cr is set, and samples exceeding this level may indicate proteinuria and glomerular or tubular injury in children and adolescents [23]. Accordingly, 21 individuals (5.25%) had a TP/Cr ratio that exceeded the cut-off value of 0.2, indicating proteinuria and possible glomerular or tubular injury. However, upon further data investigation, only 1 of the above-mentioned individuals also had an elevated NAG-to-creatinine ratio measured in this study.

### 2.5. Correlations

**Figure 1:**
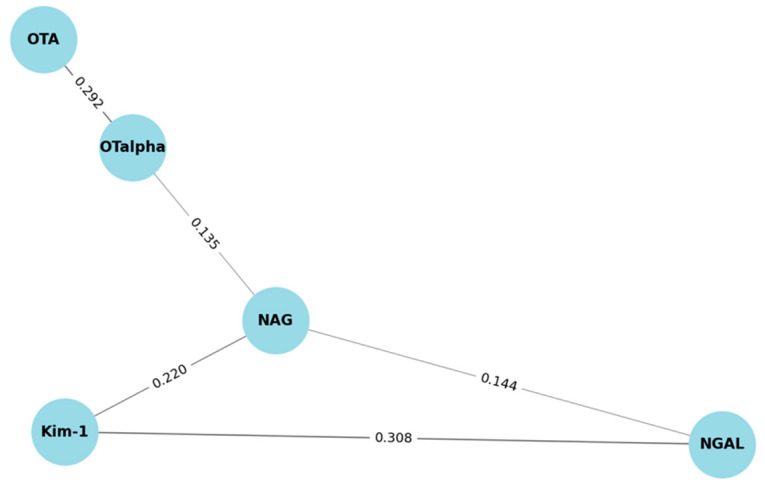
Correlation among kidney injury biomarkers and mycotoxin biomarkers.

Figure 1 shows the significant Pearson’s correlation coefficients (*p*-value <0.05) after adjusting data for normality. Accordingly, OTα was weakly positively correlated with OTA and NAG at r = 0.292 and r = 0.135, respectively. All biomarkers were weakly positively correlated with the following Pearson’s coefficient reported: NAG and NGAL with r = 0.144, NAG and Kim-1 with r = 0.220, and NGAL with Kim-1 with r = 0.308.

Table 9 summarizes the significant (*p*-value < 0.05) Spearman’s correlations between various food consumption patterns, kidney injury biomarkers, and urinary OTA and OTα levels. Overall, most of the reported correlations were weak and should be approached captiously. A few food items showed statistically weak but positive correlations, such as croissant with NGAL, brown bread with Kim-1, regular breakfast cereal with NAG, and Turkish coffee and OTα. No strong or biologically meaningful correlations were observed for the remaining food groups. Overall, the pattern of findings suggests that food-specific associations were limited in strength and do not indicate clear dietary drivers of OTA or kidney injury biomarker levels.

### 2.6. Probable OTA Dietary Intake

Data was primary fixed by identifying outliers based on skewness, kurtosis, and visual inspection of boxplots. After outlier removal, variables showed acceptable distribution, so the mean was used for PDI calculation. The probable dietary intake (PDI) was calculated using mean urinary OTA that was equal to 0.09 μg/L and average weight equal to 53.66 kg. Accordingly, the PDI obtained was equal to 0.081 μg/kg bw/day, indicating a weekly intake of 0.567 μg/kg bw.

### 2.7. Risk Assessment

**Figure 2:**
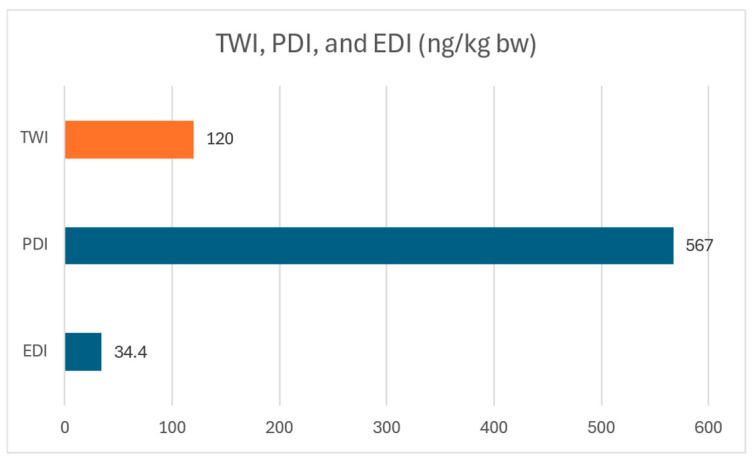
Comparison between TWI, EDI, and PDI in ng/kg bw.

According to EFSA, the tolerable weekly intake (TWI) of OTA is 120 ng/kg bw [24], meaning that the value of intake found in this study from PDI was around 4.7 times the TWI set by EFSA, indicating a high risk from OTA exposure in Lebanese children and adolescents, while the value attained from EDI indicates a safe weekly exposure below the limit of 120 ng/kg bw (Figure 2). Additionally, the margin of exposure (MOE) for neoplastic and non-neoplastic was calculated from PDI and was found to be 180 and 59, respectively, indicating a high risk from OTA exposure in children and adolescents in Lebanon (Table 10). For EDI, the neoplastic MOE indicated a high risk, as it was found to be 2947 less than 10,000, while the non-neoplastic indicated low risk, since it was equal to 961, which is higher than the limit of 200.

## 3. Discussion

The following discussion of the results mainly concentrates on four different parts: rates and implications of OTA and OTα occurrence indications; renal biomarker levels and significance; correlations with dietary intake; and public health implications. This study aimed to evaluate the levels of OTA, OTα, and kidney injury biomarkers N-acetyl-β-D-glucosaminidase (NAG), Kidney Injury Molecule-1 (KIM-1), NGAL, as well as creatinine and total protein, while correlating these findings with dietary patterns. OTA is a known toxigenic agent with main toxicity affecting the kidneys; however, its complex toxicokinetics presents a challenge in accurately assessing its impact on renal function. When ingested, OTA is absorbed in the gastrointestinal tract at the level of the stomach and the jejunum with species-dependent differences [25]. In humans, approximately 93% of ingested OTA is bioavailable, and although the specific transport mechanisms are not well-known, research suggests that through destabilizing the intestinal epithelial cell tight junctions, OTA could facilitate its own absorption [25,26]. Following absorption, OTA binds to albumin with high affinity, where 99.8% of the toxin is found in the bound form, resulting in a long half-life in the blood reaching up to 35 days [25,27]. In animals and humans, OTA can be metabolized in the kidney, liver, and intestines. The metabolism of OTA leads to the formation of a hydrolyzed derivative known as OTalpha (OTα), which is produced at the level of the gut with less potency than the parent compound; however, this biotransformation is poor and slow, which can play an important role in toxicity [28].

Both OTA and OTα undergo excretion in the kidneys, and while the excretion of the first is slow, the elimination of the latter is efficient and rapid. Accordingly, this was evident in the current study, as OTA was found in 14.2% of urine samples at a mean of 1.01 μg/L, while OTα was detected in 59.5% of urine samples with a higher mean of 3.54 μg/L. This difference in detection and mean concentration confirms that OTA is being degraded into OTα, which is more abundant in urine than its parent mycotoxin, and emphasizes its role in detecting short-term exposure to OTA while rendering it a less reliable indicator of sustained nephrotoxic effects. Additionally, this was evident in the weak positive correlation found between OTA and OTα, suggesting again a different appearance pattern in urine samples, reflecting long- or short-term exposure. Nonetheless, it is worth noting that OTα is also excreted through the fecal route; however, it was found that both OTA and OTα were found to be 2–3 times higher in urine than in feces [29], and it was reported that approximately 61% of OTα is excreted in urine and 9% in feces [30]. Additionally, when considering OTA metabolism, OTα is the most significant derivative, while other minor metabolic pathways also exist, leading to the production of different metabolites from processes like dichlorination into OTB and generation of OH-OTA derivatives in the liver by cytochrome P450 [29]. Therefore, for a comprehensive assessment of OTA exposure, other metabolites can also be evaluated in future studies. The findings of OTA and OTα in urine from this study are like those reported in Spain, where OTα was more prevalent and found in 60.6% of samples with a mean of 0.441 μg/L, whereas OTA was detected in 12.5% of samples at a mean of 0.237 μg/L [31]. In Germany, OTA was reported to be more frequent in urine than OTα, where they were found in 100% and 78% of samples at means of 0.21 and 1.33 μg/L, respectively [32].

This result again can be explained by several factors: first, the slow excretion of OTA is caused by its strong bond to albumin and long half-life, which complicates the interpretation of urinary OTA measurements. OTA levels may not reliably reflect systemic or, more critically, renal tissue exposure because OTA’s strong affinity for plasma proteins restricts its renal excretion. This bond also makes glomerular filtration negligible; instead, OTA is excreted through tubular secretion [25]. At the level of the renal tubules, OTA secretion is mediated by organic anion transporters (OAT) that uptake OTA from the blood; however, simultaneously, this transport system is responsible for the reabsorption of OTA, resulting in its accumulation in the kidney tubules and contributing to its toxicity [33,34]. Additionally, research has shown that exposure to low doses of OTA causes increased renal expression of OAT, suggesting possible health risks of chronic OTA exposure [35]. Furthermore, the low prevalence of OTA in urine, unlike OTα, can be explained by the fact that OTA is distributed and accumulates in body organs, mainly the kidney and lungs, followed by the liver and bile, spleen, heart, brain, muscle tissue, and fat [29]. Supporting this notion, animal studies have demonstrated significantly higher OTA concentrations in kidney tissue compared to urine, suggesting that urinary measurements may underestimate the actual renal burden [30].

The kidney is the main target organ for OTA toxicity, and it is susceptible mostly due to its high blood flow and special metabolism [36]. Renal damage is usually evaluated by quantifying urinary biomarkers of nephrotoxic mycotoxins, which can refer to the excreted mycotoxin or its breakdown products, such as OTα in this case. Other novel biomarkers were developed to evaluate kidney damage that, according to Ráduly et al. (2021), are defined as “parameters of physiological, chemical, structural, and genetic changes that show the severity, progress, or presence of histological alteration.” Among those biomarkers are effect biomarkers, including N-acetyl-β-D-glucosaminidase (NAG), Kidney injury molecule-1 (KIM-1), and NGAL, which are excreted in urine because of tubular injury [36]. NAG, KIM-1, and NGAL could be used to detect acute kidney injury (AKI), unlike traditional methods of blood analysis that detect kidney disease in later stages, such as estimated glomerular filtration, blood urea nitrogen, and serum creatinine [11].

AKI can start at early ages in childhood, especially with exposure to toxicants including OTA, because children exhibit low body weight, making them more susceptible to its toxic effects. Therefore, early detection of OTA, its biomarkers, and kidney injury biomarkers in urine may indicate the severity of AKI in children, allowing for early control measures [36].

In this study, the kidney injury biomarker Kim-1 was found in 86% of urine samples and the biomarkers NGAL and NAG were both found in 100% of tested samples. They were found in mean concentrations of 325.7 pg/mL, 57.4 pg/mL, and 175.3 mU/L for Kim-1, NGAL, and NAG, respectively. However, these values cannot be used to predict kidney injury unless evaluated as kidney injury markers-to-creatinine ratios. Nonetheless, it is important to note that the cut-offs suggested by the U.S. Food and Drug Administration [37] were applied to evaluate the kidney injury biomarkers-to-Cr ratio found in this study. However, no universally accepted cut-offs exist for children and adolescents, and the few studies that have proposed such values mainly included children and adolescents with preexisting health conditions. Accordingly, in this study, only six samples were found to exceed the cut-off value of 8.7 mU/mg of the NAG-to-Cr ratio, indicating that most of the participants fell within the normal range of kidney function, while only very few had elevated biomarkers of proximal tubular damage. To further assess renal injury, TP, Cr, and the TP-to-Cr ratio were evaluated; accordingly, 21 participants (5.25%) had an elevated TP-to-Cr ratio, i.e., exceeding the limit of 0.2 indicating proteinuria and possible glomerular or tubular injury. However, upon further investigation, only 1 individual of the above mentioned had an elevated NAG-to-Cr ratio; therefore, this shows that the above participants most likely had glomerular damage rather than tubular, as almost none of the biomarkers indicating tubular damage were elevated along with the TP/Cr ratio except in this one individual, implying that the damage may have been related to OTA exposure.

Since biomarker expression does not necessarily indicate renal damage due to mycotoxin exposure, a combined measurement of exposure biomarkers and effect biomarkers is recommended, as it can provide good evidence of correlation [11]. Therefore, when assessing correlations, all reported ones were found to be positive weak correlations; specifically, urinary OTα was correlated with NAG, and all kidney injury biomarkers—NAG, Kim-1, and NGAL—were correlated with one another. The correlation between OTα and NAG, although weak, suggests that OTA exposure might induce subtle renal tubular stress detectable through kidney injury biomarkers such as NAG. This also aligns with study findings that suggested an increased expression of kidney injury biomarker genes before changes in renal histopathology and in traditional kidney parameters that indicate impaired renal function [38]. This suggests the initiation of subclinical damage reflected by early kidney injury biomarkers that has not yet progressed into a full pathological condition. Beyond OTA’s inherent properties, a confluence of dietary and environmental factors may further confound the relationship between OTA exposure and kidney injury biomarkers. The potential influence of other nephrotoxic agents, such as aflatoxin B1, fumonisins, bisphenol A, and phthalates [39,40] must be considered. Particularly in regions experiencing economic instability, shifts in dietary habits may lead to increased consumption of lower-cost, potentially contaminated foods [17,18], elevating the risk of exposure to multiple nephrotoxic compounds and thereby influencing biomarker levels independently of OTA.

Despite recognizing this potential tissue-level relationship, the interpretation of kidney injury biomarkers like KIM-1, NGAL, and NAG must be approached with caution, as their elevations can be attributed to a multitude of factors unrelated to OTA exposure. For instance, KIM-1 is linked to tubular injury but can also be elevated by heavy metals and other environmental toxins [10]; NGAL is a marker of acute kidney injury and inflammation that can rise due to oxidative stress and infections [12]; and NAG, a lysosomal enzyme, is elevated in renal tubular injury but can also be affected by metabolic conditions and dietary factors [11]. While normalization using ratios such as KIM-1/total protein and NGAL/creatinine helps mitigate some variability, it does not eliminate confounding effects entirely. As such, the elevated biomarker levels observed in our study may reflect cumulative exposure to multiple renal stressors rather than a direct effect of OTA. One notable consideration is the potential role of high aspartame consumption, especially among younger populations, where soft drinks containing this artificial sweetener are common [41]. Aspartame, which contains phenylalanine, may compete with OTA for albumin binding sites and thereby promote OTA displacement and faster renal elimination [25,42]. This accelerated clearance could shorten OTA’s half-life from about 35 days to roughly 10 days and result in lower urinary OTA and OTα levels. Although this hypothesis cannot be evaluated in our study due to the absence of aspartame consumption data, it warrants investigation in future research.

Additionally, when assessing the relationship between diet patterns, mycotoxin, and kidney injury biomarkers, it was evident that alcoholic beverage consumption among participants was correlated in a positive but weak pattern with NGAL, Kim-1, and NAG. This was also shown in animal studies that reported elevation of kidney injury biomarkers, specifically Kim-1 and NGAL, in ethanol-fed mice, which might further create an additional confounding factor in participants that consume alcohol [43]. Other beverages, such as Turkish coffee and energy drinks, were also correlated in a weak positive pattern with OTα and Kim-1, respectively, suggesting renal stress related to their consumption among the study age group. On the other hand, foods specifically from the nuts group were negatively and weakly correlated with OTα, suggesting that such foods might be low in contamination. Alternatively, a possible explanation arises when considering Sergent et al.’s (2005) observation that polyphenols in food increase the absorption of OTA and its bioavailability at the level of the small intestine [44]; accordingly, nuts that are rich in polyphenols [45] might contribute to this mechanism, which leads to greater absorption of OTA at the level of the small intestine and its decreased availability in the gut for detoxification to OTα. This mechanism, which warrants further hypothesis study, is a plausible reason for the observed weak negative correlation between nuts and OTα and deserves more thorough future exploration. Additionally, there were some positive weak correlations between items from the cereal food group and certain kidney injury biomarkers, such as croissant, brown bread, and regular breakfast cereals.

Finally, in this study EDI was assessed reflecting the estimated daily intake obtained from FFQ data and previous contamination data. However, the overall estimated exposure to OTA obtained did not show a significant correlation with urinary OTA, OTα, or other kidney injury biomarkers. This might be attributable to several reasons: first, the underreporting of food consumption by participants; second, the lack of data on OTA contamination from different food sources; third, the presence of masked OTA that was not detected in previous food contamination studies; and finally, the EDI calculated in this study was achieved through considering previous contamination data from different studies done in Lebanon instead of testing real food consumed by participants. Therefore, the EDI calculated might not reflect the actual exposure in real-time data, and this limitation could be addressed in future studies. PDI was also calculated from urinary OTA data, and, accordingly, a big difference was reported between EDI and PDI, as the first was estimated to be 4.92 ng/kg bw/day while the second was reported to be 81 ng/kg bw/day. This result is in agreement with that from other studies; for example, Solfrizzo et al. (2014) reported a higher level of exposure estimated using a urinary approach rather than a dietary one and attributed that to several reasons like the ones mentioned previously in this study [46]. Similarly, Al Ayoubi et al. (2021) reported a urinary-based PDI equal to 21.73 ng/kg bw/day, while dietary-based exposure was much lower at the level of 1.4 ng/kg bw/day [47].

Assessing the risks from both EDI and PDI in this study shows that, for the estimated daily exposure using the dietary approach “EDI”, the weekly intake was shown to be less than the tolerable weekly intake (TWI) set by EFSA. However, the MOE for neoplastic effects indicates a high risk due to chronic exposure, while the MOE for non-neoplastic effects indicates a low risk. On the other hand, the PDI using the urinary approach shows levels of weekly intake almost 4.7 times higher than the TWI with MOEs, which also indicates a high risk for both neoplastic and non-neoplastic effects of OTA. Therefore, while EDI underestimates exposure due to the many reasons previously mentioned, PDI might be a better risk indicator from OTA exposure. According to Gilbert et al. [48] urinary OTA levels reflect recent intakes, and its occurrence in urine is a good indication of recent exposure; however, the relationship between OTA consumption and excretion needs to be further studied. Hence, this signifies that at current levels of exposure, Lebanese children and adolescents are at a higher risk for developing chronic and acute complications. And while this study did not establish a direct and high correlation from the consumption of cereals and other affordable food items to OTA-caused renal damage, it is important to recognize that this study included a limitation, namely that current contamination in food was not measured; therefore, the true effect of consumption of such food on renal health of children and adolescents in Lebanon may have been underestimated. Additional limitations that can be addressed in future studies include the presence of confounding factors that prohibited the establishment of a direct relationship between OTA and renal damage in this study, the recall bias in the FFQ that might have affected the consumption data, and the lack of new contamination data after the financial crisis in Lebanon. Additionally, the PDI approach as reported by Gilbert et al. should be approached with caution and should be further studied to evaluate the suitability of the relationship between OTA and PDI [48] Therefore, given those limitations, it is important to note that certain associations might have occurred by chance, so such data need to be approached with caution and confounding factors need to be further addressed in future studies.

Moving forward, future research efforts should focus on several key areas. First, they should address the measurement of OTA in blood or plasma alongside OTα and other biomarkers in urine that might provide a more accurate assessment of systemic exposure and its correlation with kidney injury. Given that OTA’s high affinity for plasma proteins means that most circulating OTA is bound in the bloodstream rather than freely excreted in urine [9], plasma or blood measurements, which capture both free and protein-bound fractions, offer a more comprehensive evaluation of total systemic exposure. Blood sampling offers a more stable matrix for detecting chronic exposure. Studies have indicated that measuring OTA in blood or plasma is more reflective than measuring urinary OTA, which reflects short-term exposure [30]. Second, research should expand biomarker panels to include markers such as β2-microglobulin and cystatin C, which may help differentiate OTA-specific nephrotoxicity from other renal aggressors. Third, longitudinal studies that monitor biomarker and OTA levels over time are needed to evaluate the cumulative effects of chronic exposure. By adopting a comprehensive approach that integrates multiple biomarkers and dietary assessments, while accounting for the contribution of other nephrotoxicants, future investigations can more specifically define the mechanisms by which OTA contributes to kidney injury. This could include the identification of novel biomarkers of OTA exposure, such as specific urinary and plasma metabolites or molecular signatures, using advanced omics technologies to differentiate OTA-related effects from those of other nephrotoxicants, or the development of non-invasive biomarkers that better reflect tissue-specific toxicity, ultimately informing the development of more effective strategies for mitigating the risks associated with OTA exposure. Finally, the development of control and monitoring strategies of OTA in Lebanon is of utmost importance; this includes adopting good agricultural practices and good storage practices in addition to performing surveillance studies to detect contamination in the Lebanese market. Control also includes applying strict measures upon food admission at the borders, since most of the foods available on the Lebanese market are imported products.

## 4. Conclusions

As OTA risk is well-established, this is the first study of its kind to be performed that reports the first data from Lebanon on food contamination and its relationship to kidney injury in a defined Lebanese age group. In this study, OTA exposure was detectable both through FFQ and contamination studies, and the presence of urinary OTA and OTα was evaluated. The reported PDI levels from urinary OTA indicated a high risk to children and adolescents from current exposure levels. Given this finding, the kidney injury biomarkers reported, even though at low levels and at low correlation levels with OTA exposure, suggested the onset of subclinical damage that had not yet progressed into a full pathological condition. Therefore, according to the findings from the current study, it is crucial to adopt serious measures to lower OTA exposure in such a vulnerable age group, specifically by maintaining OTA in food at levels that are as low as reasonably achievable in nationally produced and imported foods. Such measures include applying strict control through the whole food chain, good agricultural practices, good storage practices, and continuous surveillance and monitoring.

## 5. Materials and Methods

### 5.1. Study Design and Data Collection

**Figure 3:**
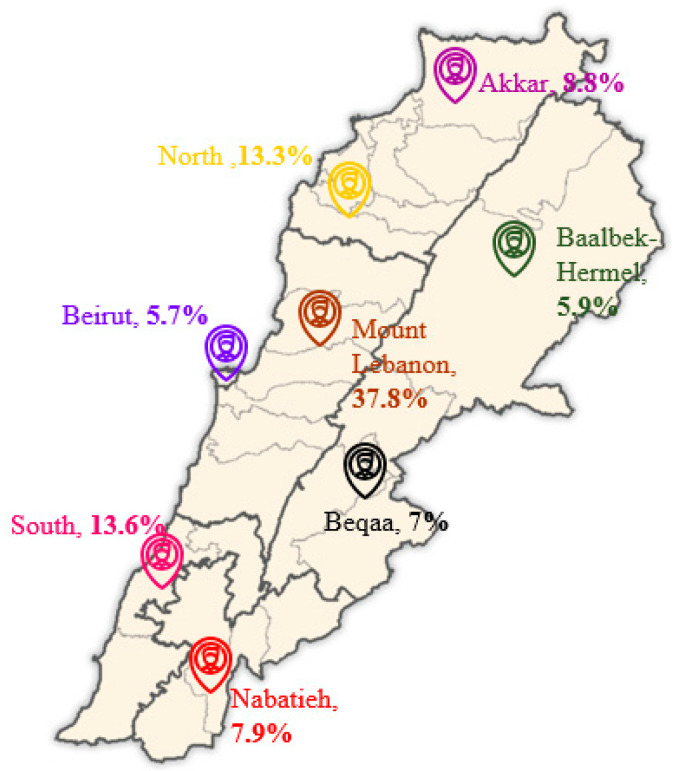
Distribution of study participants across governorates (https://commons.wikimedia.org/wiki/File:Lebanon_districts.png accessed: 25 May 2024).

A cross-sectional survey, including a nationally representative sample of Lebanese school-aged children and adolescents, was conducted between December 2022 and March 2023 in Lebanon. The probability cluster sampling method was used to select study participants, who were recruited from the eight Lebanese governorates: Mount Lebanon, Beirut, South Lebanon, North Lebanon, Akkar, Beqaa, and Baalbek. The sample size was calculated using the standard single-proportion formula (*n* = [*p*(1 − *p*)] × (*Zα/*2^2^*/e*^2^)). A conservative estimate of *p* = 0.5 was used to maximize the required sample size. Due to feasibility and resource constraints, a minimum of 400 participants were targeted, which was anticipated to provide sufficient power for the primary correlational analyses. Considering a 10% non-response rate, we reached a total of 442 Lebanese adolescents participants. Participant distribution across the governorates is shown in Figure 3.

### 5.2. Ethical Considerations

The study was carried out according to the Helsinki Declaration’s ethical guidelines. The Ethics Committee of the Al-Zahraa University Medical Center, Beirut, Lebanon (Reference Nb 10-12-2022) provided their ethical approval to conduct this study. A written consent form was obtained from parents of participants.

### 5.3. Inclusion and Exclusion Criteria

To ensure relevance to the goals of the study, participants were recruited based on the following eligibility criteria: All the participants had to be Lebanese, aged between 10 and 18 years, and free of chronic diseases. Moreover, only one adolescent child was recruited from each household.

### 5.4. Data Collection

Data were collected by skilled dietitians who were trained prior to data collection. The overall survey took around forty-five minutes during which sociodemographic data, frequency of consumption, and anthropometric data were collected. Information was gathered through face-to-face interviews either with adolescents or their caregivers, primarily mothers for younger participants.

#### 5.4.1. Sociodemographic, Medical, and Anthropometric Data

Sociodemographic characteristics (including age, residency, educational level, primary caregiver, caregivers’ education level, and working status) and medical characteristics (such as supplement use) were collected through a questionnaire. As for weight and height, the trained dietitians measured them twice for each participant, and the average value was computed.

#### 5.4.2. Food Frequency Questionnaire (FFQ)

The dietary intake of participants was assessed using a 119-item semi-quantitative food frequency questionnaire (FFQ) (Supplementary Files: Adolescents FFQ), previously validated among Lebanese adolescents [8]. Additional items, usually consumed at least once a week by the participants, were taken into consideration. The FFQ captured the frequency of consuming various foods over the previous year, with interviewees recording servings, grams, and consumption frequency (daily, weekly, or monthly). Weekly consumption was transformed to daily by dividing the value by 7, monthly consumption was transformed into daily consumption by dividing the value by 30, and all values were unified as daily consumption in grams per day (g/d). Visual aids and instructions were provided to assist participants in accurately recalling food intake and estimating portion sizes.

#### 5.4.3. 24-Hour Recalls

Three non-consecutive 24 h recalls (24HR) were administered: two during a typical weekday and another one during the weekend. The average of the three days in g/d was computed to get the consumption of items mentioned by the participants. Visual aids were provided to assist participants in recalling and estimating portion size as accurately as possible.

#### 5.4.4. Average Consumption

Food items identified in 24HR were classified according to the FFQ food groups, and the average consumption of a participants was calculating using the following formula:

$$\mathrm{Average}\;\mathrm{consumption}\;=\;\frac{\left[g\;\left(FFQ\right)\;+\;g\;\left(24HR\right)\right]}{2}$$

The overall adolescent population was classified into two age categories—10–13 years and 14–18 years—to compare the nutrient intake to the DRI.

#### 5.4.5. Food Contamination Data

Food contamination data were extracted from “Mycotoxins in Lebanese Food Basket: Database on Occurrence and Exposure” [21], which includes a compilation of mycotoxin occurrence studies published in Lebanon between 2004 and 2022. Accordingly, the mean, standard deviation and minimum and maximum contamination of food with OTA were collected and used in the assessment of adolescents’ dietary exposure according to their reported consumption patterns.

#### 5.4.6. Dietary Exposure Calculation

The dietary exposure to OTA for each participant was calculated according to the following equation:

$$\mathrm{EDI}\;(\mathrm{ng}/\mathrm{kg}\;\mathrm{bw}/\mathrm{day})\;=\;\frac{DI\;\left(\mathrm{kg}/\mathrm{day}\right)\;\times \;MC\;\left(\mathrm{ng}/\mathrm{kg}\right)}{Body\;weight\;\left(\mathrm{kg}\right)}\;\;\;\;\;\;\;\;\;\;\;$$

where EDI stands for the estimated daily intake, DI for food daily intake, and MC for OTA mean contamination in the selected food. Next, the participants’ average daily exposure to OTA was calculated.

#### 5.4.7. Urine Samples

A total of 400 samples were collected from participants’ first urine of the day. The samples were collected in sterile urine plastic cups, placed in ice containers, and then directly transferred to the lab and stored at −20 °C.

### 5.5. Materials and Chemicals

Standards of OTA (50 μg/mL in Benzene: Acetic Acid 99:1) were purchased from Supelco (Bellefonte, PA, USA), and standards of OTα were purchased from HPLC Standards GmbH (Cunnersdorf, Germany). β-Glucuronidase/Arylsulfatase enzyme vials were purchased from Roche (Basel, Switzerland). Methanol, ascorbic acid, acetic acid, phosphoric acid, propanol, chloroform, EDTA, and water (HPLC grade) were purchased from Sigma-Aldrich (Steinheim, Germany).

ELISA kits Human Lipocalin-2 (NGAL), Human Kim-1, and N-Acetylglucosaminidase (beta-NAG) Activity Assay kits were purchased from Abcam (Cambridge, UK).

### 5.6. ELISA Tests

#### 5.6.1. Human Lipocalin-2 (NGAL)

Standards and samples were prepared, 100 µL of diluted sample were added to appropriate wells, and then the plate was sealed and incubated at 37 °C for 90 min. Next, the contents were discarded. Then 100 µL of biotinylated anti-human lipocalin-2 antibody was added into each well and the plate was sealed and incubated at 37 °C for 90 min. After that, the plate was emptied and washed with PBS, and 100 µL of Avidin-Biotin-Peroxidase Complex was added to each well. Then the plate was sealed and incubated at 37 °C for 30 min. Next, the plate was emptied and washed with PBS, 90 µL of TMB color-developing agent were added into each well, and the plate was sealed and incubated at 37 °C in the dark for 25 min. Finally, TMB Stop Solution was added into each well and the absorbance was read directly at 450 nm.

#### 5.6.2. Human Kim-1

Samples, standards, and essay buffers were added as indicated by the manual to the plate and it was sealed and incubated for 30 min at room temperature with mixing at 300 rpm. Then the contents were emptied, and the plate was washed. Next, 100 µL of antibody were added to the wells as indicated and the plate sealed and incubated for 30 min at room temperature with mixing at 300 rpm. Then the plate was emptied and washed before adding 100 µL Conjugate to the indicated wells. The plate was then sealed and incubated for 30 min at room temperature with mixing at 300 rpm. After that, the plate was emptied and washed, then 100 µL of TMB solution were added into each well and the plate was sealed and incubated for 20 min at room temperature with mixing at 300 rpm. Finally, 100 µL Stop Solution was added to each well, and the absorbance was directly read at 450 nm.

#### 5.6.3. N-Acetylglucosaminidase (Beta-NAG) Activity Assay (NAG)

Standards, samples, and positive controls were added to different wells as indicated in the manual. Then 55 µL of NAG Substrate were added, followed by 55 µL of NAG Assay Buffer, and the plate was mixed and incubated at 37 °C for 30 min. After that, 25 µL of NAG Stop Solution was added to the wells, and the plate was incubated at 37 °C for 10 min. Finally, absorbance was measured at 400 nm.

### 5.7. HPLC Quantification of OTA and OTα

#### 5.7.1. Samples Preparation

Sample preparation was conducted according to a method reported by Muñoz et al. (2010) [49]. First, 3 mL of the urine samples were mixed with 0.25 mL hydrolysis buffer (13.6 g sodium acetate hydrate, 1 g ascorbic acid, 0.1 g EDTA in 100 mL deionized water, adjusted to pH 5.0 with acetic acid) and 40 µL of β-Glucuronidase/Arylsulfatase enzyme and were incubated at 37 °C for 24 hrs. Next, liquid–liquid extraction was performed. The incubated samples were mixed with 3 mL 1% NaHCO_3_ in water, and the pH was adjusted with 1 M phosphoric acid in a pH range between 3 and 4. The mixture was then centrifuged at 4500 rpm for 15 min. The aqueous upper layer was aspirated with a pipette and discarded; then, 2 mL of the organic layer was evaporated under a nitrogen stream at 45 °C. Th extract was then reconstituted in 1200 µL of methanol/water (1:1, *v*/*v*) and filtered through a 0.45 µm syringe filter.

#### 5.7.2. HPLC Analysis

HPLC analysis was conducted according to a method reported by Muñoz et al. (2010) [49]. Reverse-phase HPLC (Waters 2690^®^, Waters Corp., Milford, MA, USA) coupled with a fluorescence detector (Waters 2475^®^) and a (Supelco Discovery^®^) HS C18 column (250 mm × 4.6 mm I.D., 5 μm particle diameter) fitted with a C18 guard column (Supelco Supelguard^®^, Sigma-Aldrich Co., St. Louis, MI, USA) at 40 °C temperature was used for analysis. Excitation and emission wavelengths were 333 and 450 nm, respectively. Two mobile phases were used to achieve a gradient. Phase A consisted of acetic acid 2%: methanol (63:34), and phase B was methanol:isopropanol (90:10). The gradient was as follows: 0–15 min 95% A, 15–16 min 95–60% A, 16–30 min 60% A, 30–31 min 60 to 5% A, 31–33 min 5% A, 33–34 min 5–95% A, 34–45 min 95% A. Flow-rate was 1 mL/min and injection volume was 100 µL. Retention time for OTA was 28 min, whereas for OTα it was 12 min. A calibration curve was constructed with R^2^ equal to 0.9998 and 0.9985 for OTA and OTα, respectively. The method performance parameters included the limit of detection (LOD) and the limit of quantification (LOQ), which were measured according to signal-to-noise ratios (S/N) of 3:1 and 6:1, respectively. The LOD and LOQ were reported to be 0.028 and 0.08 μg/L, respectively for OTA and 0.04 and 0.121 μg/L, respectively, for OTα.

### 5.8. Interpretation of OTA Exposure Risk

#### 5.8.1. Probable Daily Intake

According to Solfrizzo et al. (2014) [46] probable daily intake (PDI) of OTA can be calculated using the urinary OTA value according to the following formula:

$$\mathrm{PDI}\;(\mathrm{ng}/\mathrm{kg}\;\mathrm{bw}/\mathrm{day})\;=\;\frac{C\;\times \;V\;\times \;100}{W\;\times \;E}$$

where C: OTA urinary concentration

V: mean 24 h human urine volume (1.5 L)

W: mean body weight

E: mean urinary excretion of mycotoxins, where it is considered to be 2.6% for OTA according to Solfrizzo et al. (2014) [46]

#### 5.8.2. Margin of Exposure

Margin of exposure (MOE) is proposed as an assessment tool for the risk assessment of genotoxic and carcinogenic substances in food, such as OTA [50]. Accordingly, to assess the risk from OTA exposure both from the dietary intake estimated in this study and from urinary OTA biomarker estimated probable intake, the following formula was used:

$$\mathrm{MOE}\;=\;\frac{BMDL10\;}{EDI/PDI\;}$$

where BMDL10 stands for the benchmark dose lower confidence limit, EDI for “Estimated Daily Intake” obtained from dietary recall and contamination studies, and PDI for probable dietary intake obtained from the urinary OTA biomarker.

For BMDL10, EFSA established two values—one that is used to assess critical neoplastic effects related to kidney cancer, and the other for chronic non-neoplastic effects related to nephrotoxicity and kidney damage, which are equal to 14.5 and 4.73 μg/kg bw/day, respectively [24]. Accordingly, MOE neoplastic (MOEneo) ≥ 10,000 or MOE non-neoplastic (MOE non-neo) ≥ 200 indicates that the exposure is of low health concern [24].

#### 5.8.3. Weekly Exposure to OTA

The provisional weekly tolerable intake (PTWI) set by EFSA at 120 ng/kg bw/day was used to further evaluate the risk of kidney disease from OTA exposure [24]. The EDI and PDI values attained were used to compare the reported exposure in this study to the PTWI.

### 5.9. Interpretation of Kidney Injury Biomarkers Results

Because kidney injury biomarker values cannot be used to evaluate kidney health in the absence of a clear cut-off value, the ratio of kidney injury biomarkers to creatinine is usually used, and this was applied uniformly to all three kidney injury biomarkers. In this study, as in many others, spot urine samples were collected due to convenience, while generally the preferred method is 24 h urine collection. This method creates a challenge due to several factors, including the time of urine collection, its concentration, and its flow rate. Therefore, according to Tang et al. (2015), in such cases the biomarkers obtained are usually normalized to creatinine concentration to account for those differences [51]. Additionally, this helps create a factor to compare with established cut-offs according to a defined biomarker-to-creatinine ratio. While normally there is no unified cut-off value for the biomarker-to-creatinine ratio, as it differs according to studies in different countries and on different ethnicities, in this study the following cut-off values were used: 7.6 ng/mg Cr for Kim-1, 9.6 ng/mg Cr for NGAL, and 8.7 mU/mg Cr for NAG. These were proposed to be medically significant cut-off values by the U.S. Food and Drug Administration [37].

### 5.10. Statistical Analysis

All statistical analyses were performed using SPSS 19.0 (SPSS Inc., Chicago, IL, USA). Means were computed in addition to correlations using both Pearson’s and Spearman’s correlation according to data normality with significance at less than 0.05. Normality was assessed using skewness and kurtosis, in which data were considered normal when they were between −2 and +2. Correlation was tested between different biomarkers, urinary OTA, OTα, and dietary intakes. *p*-values reported in Table 1, Table 2, Table 3 and Table 4 were generated using the appropriate statistical tests: the chi-square test for categorical variables and the independent samples *t*-test for normally distributed continuous variables (based on skewness and kurtosis). When normality assumptions were not met, the Mann–Whitney U test was applied.

## Figures and Tables

**Table 1:** Demographic and sociodemographic characteristics of the study population, overall and by gender.

		Overall (n = 442) N (%)	Male (n = 196) N (%)	Female (n = 246) N (%)	*p*-Value
Age category	10–13 years	172 (38.9%)	95 (48.5%)	77 (31.3%)	<0.001 *
	14–18 years	270 (61.1%)	101 (51.5%)	169 (68.7%)	
Residence	Mount Lebanon	167 (37.8%)	85 (43.4%)	82 (33.3%)	0.588
	Beirut	25 (5.7%)	6 (3.1%)	19 (7.7%)	
	South Lebanon	60 (13.6%)	20 (10.2%)	40 (16.3%)	
	North Lebanon	59 (13.3%)	19 (9.7%)	40 (16.3%)	
	Akkar	39 (8.8%)	15 (7.7%)	24 (9.8%)	
	Nabatieh	35 (7.9%)	20 (10.2%)	15 (6.1%)	
	Beqaa	31 (7.0%)	14 (7.1%)	17 (6.9%)	
	Baalbek-Hermel	26(5.9%)	17 (8.7%)	9 (3.7%)	
Primary caregiver (n = 440)	Mother only	15 (3.4%)	7 (3.5%)	8 (3.2%)	0.255
	Father only	8 (1.8%)	4 (2.05%)	4 (1.6%)	
	Both parents	407 (92.5%)	182 (93.3%)	225 (91.8%)	
	Others	10 (2.3%)	2 (1.02%)	8 (3.2%)	
Education level	Elementary school level (grades 1–6)	112 (25.3%)	61 (31.1%)	51 (20.7%)	<0.001 *
	Intermediate school level (grades 7–9)	119 (26.9%)	67 (34.2%)	52 (21.1%)	
	Secondary school level (grades 10–12)/vocational	122 (27.6%)	48 (24.5%)	74 (30.1%)	
	University level	89 (20.1%)	20 (10.2%)	69 (28%)	
	Illiterate	0 (0.0%)	0 (0.0%)	0 (0.0%)	
Mother education level	Elementary school level (grades 1–6)	72 (16.3%)	30 (15.3%)	42 (17.1%)	0.707
	Intermediate school level (grades 7–9)	106 (24%)	45 (23%)	61 (24.8%)	
	Secondary school level (grades 10–12)/vocational	106 (24%)	48 (24.5%)	58 (23.6%)	
	University level	140 (31.7%)	64 (32.7%)	76 (30.9%)	
	Illiterate	18 (4.1%)	9 (4.6%)	9 (3.7%)	
Father education level	Elementary school level (grades 1–6)	101 (22.9%)	45 (23%)	56 (22.8%)	0.822
	Intermediate school level (grades 7–9)	104 (23.5%)	40 (20.4%)	64 (26%)	
	Secondary school level (grades 10–12)/vocational	105 (23.8%)	57 (29.1%)	48 (19.5%)	
	University level	113 (25.6%)	46 (23.5%)	67 (27.2%)	
	Illiterate	19 (4.3%)	8 (4.1%)	11 (4.5%)	
Working status (n = 441)	No	413 (93.7%)	180 (92.3%)	233 (94.7%)	0.314
	Yes	28 (6.3%)	15 (7.7%)	13 (5.3%)	

* *p*-value < 0.05 is significant.

**Table 2:** Health characteristics of the study population, overall and by gender.

Variable		Overall (442)		Male (n = 196)		Female (n = 246)		*p*-Value
		Mean	SD	Mean	SD	Mean	SD	
Anthropometric values								
Weight (kg)		53.66	16.08	54.61	18.83	52.91	13.49	0.289
Height (cm)		157.57	12.37	159.44	14.83	156.09	9.76	0.007 *
BMI (kg/m^2^)		21.24	4.52	20.95	4.87	21.47	4.22	0.234
		**N**	**%**	**N**	**%**	**N**	**%**	***p*-Value**
BMI classification	Underweight	20	4.5%	15	7.7%	5	2%	0.072
	Healthy	304	68.8%	120	61.2%	184	74.8%	
	Overweight	64	14.5%	25	12.8%	39	15.9%	
	Obesity and severe obesity	54	12.2%	36	18.4%	18	7.3%	

* *p*-value < 0.05 is significant.

**Table 3:** Food groups consumed by Lebanese school children and adolescents, overall and by gender.

Food Groups	Dietary Intake						
	Overall (n = 446)		Female (n = 246)		Male (n = 196)		
	Mean±	SD	Mean±	SD	Mean±	SD	*p*-Value
Bread, cereals, and grains (g/d)	333.98±	163.25	296.01±	139.34	381.62±	178.27	<0.001 *
Legumes (g/d)	72.27±	58.69	72.53±	57.30	71.95±	60.53	0.919
Nuts and seeds (g/d)	44.56±	224.78	35.22±	55.65	56.28±	331.86	0.381
Vegetables (g/d)	236.64±	148.77	249.24±	157.26	220.82±	136.14	0.046 *
Starchy vegetables (g/d)	74.67±	77.25	72.91±	57.61	76.87±	96.53	0.593
Dairy products (g/d)	205.28±	177.28	183.83±	179.82	232.19±	170.71	0.004 *
**Meat and Meat Products, Poultry, Fish, Eggs**							
Red meat (g/d)	29.86±	32.51	27.31±	29.15	33.06±	36.12	0.065
Processed meat (g/d)	6.93±	14.17	6.20±	11.11	7.85±	17.25	0.225
Poultry (g/d)	34.52±	35.43	33.18±	30.91	36.20±	40.41	0.375
Fish (g/d)	6.89±	22.04	4.91±	8.63	9.38±	31.53	0.055
Eggs (g/d)	30.07±	45.67	22.69±	26.94	39.34±	60.41	<0.001 *
**Fruits, Total**							
Fruits (g/d)	253.62±	192.07	260.62±	202.76	244.83±	177.86	0.391
Fresh juices (100% fresh juices) (mL/d)	50.77±	74.86	53.58±	81.04	47.23±	66.34	0.376
**Sweets and Added Sugars**							
Sweets (g/d)	94.51±	70.76	89.35±	69.68	100.97±	71.76	0.086
Added sugars, jams, honey, and molasses (g/d)	21.45±	22.42	18.43±	14.96	25.23±	28.81	0.003 *
Hot beverages (mL/d)	163.15±	168.96	167.41±	169.85	157.80±	168.11	0.553
Non-alcoholic beverages (mL/d)	142.37±	173.67	129.62±	172.64	158.36±	174.07	0.084
Alcoholic beverages (mL/d)	2.56±	23.60	2.08±	24.04	3.15±	23.09	0.637
Added fats and oils (g/d)	59.38±	43.37	55.26±	27.76	64.56±	56.90	0.037 *

* *p*-value < 0.05 is significant; Abbreviations: SD standard deviation, n number of participants, g/d gram per day, mL/d.

**Table 4:** Food groups consumed by Lebanese school children and adolescents, by age.

Food Groups	Dietary Intake				
	10–13 Years (n = 172)		14–18 Years (n = 270)		*p*-Value
	Mean±	SD	Mean±	SD	
Bread, cereals, and, grains (g/d)	337.06±	154.95	332.01±	168.58	0.752
Legumes (g/d)	70.44±	57.57	73.44±	59.47	0.602
Nuts and seeds (g/d)	30.38±	58.62	53.59±	283.62	0.290
Vegetables (g/d)	217.92±	139.28	248.56±	153.59	0.031 *
Starchy vegetables (g/d)	70.31±	78.21	77.44±	76.65	0.345
Dairy products(g/d)	225.32±	192.54	192.51±	165.95	0.058
**Meat and Meat Products, Poultry, Fish, Eggs**					
Red meat (g/d)	26.65±	26.93	31.91±	35.51	0.098
Processed meat (g/d)	4.91±	8.38	8.22±	16.74	0.006 *
Poultry (g/d)	31.33±	38.34	36.55±	33.36	0.131
Fish (g/d)	4.19±	6.63	8.61±	27.59	0.012 *
Eggs (g/d)	31.30±	54.71	29.29±	38.92	0.653
**Fruits, Total**					
Fruits (g/d)	262.38±	194.15	248.03±	190.87	0.444
Fresh juices (100% fresh juices) (mL/d)	49.52±	79.53	51.56±	71.88	0.780
**Sweets and Added Sugars**					
Sweets (g/d)	93.11±	65.77	95.40±	73.88	0.740
Added sugars, jams, honey, and molasses (g/d)	17.91±	14.95	23.70±	25.87	0.003 *
Hot beverages (mL/d)	129.80±	123.37	184.39±	189.63	<0.001 *
Non-alcoholic beverages (mL/d)	144.47±	184.10	141.02±	167.02	0.839
Alcoholic beverages (mL/d)	.00±	.00	4.18±	30.11	0.023 *
Added fats and oils (g/d)	59.35±	51.10	59.40±	37.74	0.990

* *p*-value < 0.05 is significant; Abbreviations: SD standard deviation, n number of participants, g/d gram per day, mL/d.

**Table 5:** Exposure to OTA calculated by estimated dietary intake and compiled data on OTA contamination from research studies in Lebanon.

Food Group	Dietary Intake (g/day)	Mean OTA (μg/kg)	EDI (ng/kg bw/day)
Cereals and cereal-based products	231.85	0.85	3.68
Nuts and oilseeds	11.26	0.17	0.03
Legumes and pulses	59.63	0.03	0.03
Milk and dairy products	110.85	-	-
Fruits and fruit products	16.38	0.08	0.02
Herbs, spices, and condiments	4.94	2.87	0.26
Desserts and snacks	56.21	0.33	0.35
Stimulant beverages	54.84	0.51	0.52
Non-alcoholic beverages	0.65	-	-
Alcoholic beverages	2.09	0.87	0.03
Total			4.92

**Table 6:** Mycotoxin biomarkers mean in total samples.

	Mycotoxin Biomarker	
	OTα (μg/L)	OTA (μg/L)
Positive samples	238 (59.5%)	57 (14.2%)
Mean	3.54	1.01

**Table 7:** Kidney injury biomarkers mean in total samples and their ratio to urinary creatinine.

	Kim-1 (pg/mL)	NGAL (pg/mL)	NAG (mU/L)
Positive samples n (%)	344 (86%)	400 (100%)	400 (100%)
Mean	325.7	57.4	175.3
Ratio to urinary Cr	0.38 ng/mg Cr	0.08 ng/mg Cr	1.71 mU/mg Cr
>Cut-off	0	0	6 (1.5%)

**Table 8:** Renal function indicators presented by TP, Cr, and TP/Cr ratio.

	**Total Protein (mg/dL)**	**Creatinine (mg/dL)**	**TP/Creatinine**
Mean	11.6	124.4	0.11
>Cut-off	-	-	21 (5.25%)

**Table 9:** Significant correlations (*p*-value < 0.05) between kidney injury biomarkers and mycotoxin biomarkers with reported food products consumption.

NGAL	Beans	0.133
	Croissant	0.131
	Beer	0.215
	Wine	0.144
	Liquor/whiskey/vodka/gin/rum	0.162
Kim-1	Brown bread	0.181
	Green peas	0.159
	Energy drinks	0.139
	Liquor/whiskey/vodka/gin/rum	0.151
NAG	Regular breakfast cereal	0.163
	Canola oil	0.186
	Beer	0.169
	Liquor/whiskey/vodka/gin/rum	0.134
OTα	Peanuts	−0.150
	Almonds	−0.176
	Walnuts	−0.180
	Cake	−0.134
	Chocolate bar	−0.138
	Corn oil	0.148
	Canola oil	−0.158
	Turkish coffee	0.190

**Table 10:** Weekly intake of OTA, MOE neoplastic, and MOE non-neoplastic according to EDI and PDI values.

		Weekly Intake (ng/kg bw)	MOE Neoplastic	MOE Non-Neoplastic
PDI (ng/kg bw/day)	81	567	180	59
EDI (ng/kg bw/day)	4.92	34.44	2947	961

## Data Availability

The data presented in this study are available on request from the corresponding author. (The data are not publicly available due to privacy restrictions).

## Supplementary Materials

The following supporting information can be downloaded at: https://www.mdpi.com/article/10.3390/toxins17120577/s1.
