# Supplementary material for: Assessing Kidney Injury Biomarkers and OTA Exposure in Urine of Lebanese Adolescents Amid Economic Crisis and Evolving Dietary Patterns
Source: Toxins (Basel). 2025 Nov 30;17(12):577. doi: 10.3390/toxins17120577 (PMC12737349; doi:10.3390/toxins17120577)
Supplement: Supplementary file 1 [file toxins-17-00577-s001.zip › ADOLESCENTS FFQ En_adjusted.pdf]

FOOD FREQUENCY QUESTIONNAIRE

Think about your eating patterns during the past year while answering this questionnaire. Please indicate your usual intake of each of the following food items per Day, Week, or Month.
For example: Apple. If you consume 3 apples daily, write 3 in the “Day” column, if you think you average 3 apples a week over the year, write 3 in the “Week” column. However, if you rarely consume a food, let’s say once or twice a year, then tick below “Rarely/Never”.
Please be precise as much as you can.
Remember! The accuracy of the study results depends on the accuracy of your answers.

Food item	Serving size	Day	Week	Month	Rarely / Never
**Example: Apple**	1 item		3		
**Bread and Cereals**					
1. White bread	1 slice (30g)				
2. Brown or whole wheat bread	1 slice				
3. Breakfast cereals, regular	1 cup				
4. Breakfast cereals, bran	1 cup				
5. Rice, white, cooked	1 cup				
6. Pasta, plain, cooked	1 cup				
7. Wheat, whole, cooked / Bulgur	1 cup				
**Dairy products**					
8. Low-fat milk (2% fat)	1 cup				
9. Whole fat milk	1 cup				
10. Fat free / low fat yogurt	1 cup				
11. Whole fat yogurt	1 cup				
12. Cheese regular	1 slice (30g)				
13. Cheese low fat	1 slice (30g)				
14. Labneh	2 Tbsp				
**Fruits & Juices**					
15. Citrus Orange (1 item)	1 serving				
16. Grapefruit (1/2 item)	1 serving				
17. Peach (1 medium)	1 item				
18. Plums (2 small)	1 item				
19. Mango (1/2 item)	1 item				
20. Pineapple (3/4 cup)	1 item				
21. Melon (1 slice)	1 item				
22. Pear (1 small)	1 item				
23. Apricots (4)	1 item				
24. Loquat (12)	1 item				
25. Prickly pear (الصبير) (2)	1 item				
26. Deep Yellow or orange : Others	1 item				
27. Strawberry (12)	1 cup				
28. Grapes (32)	1 cup				
29. Others: Banana, medium	1 item				
30. Apple, fresh, small	1 item				
31. Dried fruits: raisins (2 Tbsp)	1 serving				

32. Dried fruits: dates (2)	1 serving				
33. Dried fruits: apricots (4)	1 serving				
34. Fresh fruit juice	1 cup				
35. Fruit drinks: canned/bottled	1 cup				
**Vegetables**					
36. Lettuce	1 cup				
37. Celery	1 cup				
38. Green peppers	1 cup				
39. Cucumber	1 cup				
40. Spinach	1 cup				
41. Hindbeh	1 cup				
42. Carrots	1 cup				
43. Watercress (Baqle)	1 cup				
44. Thyme	1 cup				
45. Green onion (بصل أخضر)	1 cup				
46. Mint (نعناع)	1 cup				
47. Dark green or deep yellow vegetables : others	1 cup				
48. Tomatoes, fresh, medium	1 item				
49. Corn cooked	1 cup				
50. green peas, cooked	1 cup				
51. potato, baked / boiled / mashed (1 Medium)	1 item				
52. Squash, summer (kussa) /cooked (4 medium)	1 cup				
53. Eggplant /cooked (4 medium)	1 cup				
54. Cabbage	1 cup				
55. Cauliflower	1 cup				
56. Broccoli	1 cup				
**Meat & Alternates**	**Serving size**	**Day**	**Week**	**Month**	**Rarely / Never**
57. Legumes: lentils cooked	1 cup				
58. Legumes: broad beans cooked	1 cup				
59. Legumes: Chickpeas cooked	1 cup				
60. Legumes: cowpea	1 cup				
61. Legumes: others cooked	1 cup				
62. Nuts and seeds: peanuts	1 cup				
63. Nuts and seeds: almonds	1 cup				
64. Nuts and seeds: sunflower seeds	1 cup				
65. Nuts and seeds: others	1 cup				
66. Red Meat	1 item (3 oz.)				
67. Poultry	1 item (3 oz.)				
68. Fish, (including Tuna)	1 serving (3 oz.)				
69. Eggs, whole, large	1 item				
70. Organ Meats(Liver, kidneys, brain)	1 cup				
71. Luncheon meats: Mortadell	1 slice (20g)				
72. Luncheon meats: Jambon	1 slice (20g)				
73. Luncheon meats: salami	1 slice (20g)				
74. Luncheon meats: Turkey	1 slice (20g)				

75. Luncheon meats: others	1 slice (20g)				
76. Sausages (1)	1 item (30g)				
77. Makanek (2)	1 item (30g)				
78. Hot dogs (1)	1 item (30g)				
**Fats and oils**					
79. Oil: Corn	1 Tbsp				
80. Oil: sunflower	1 Tbsp				
81. Oil: soy	1 Tbsp				
82. Oil: olive	1 Tbsp				
83. Olives (8 noires-10 vertes)	1 item				
84. Butter	1 Tbsp				
85. Ghee	1 Tbsp				
86. Mayonnaise	1 Tbsp				
**Sweets &Desserts**					
87. Cake	1 item				
88. Cookies	1 item				
89. Doughnut	1 item				
90. Muffin	1 item				
91. Croissants	1 item				
92. Ice cream	1 cup				
93. Chocolate bar	1 item				
94. Sugar	1 Tbsp				
95. Honey	1 Tbsp				
96. Jam	1 Tbsp				
97. Molasses	1 Tbsp				
98. Arabic sweets	1 item (40g)				
99. Baklawa(2)	1 item (40g)				
100. maamoul(1large)	1 item (40g)				
101. Knefeh (1)	1 item (40g)				
**Beverages**					
102. Soft drinks, regular (1 can = 1½ cup)	1½ cup (11 fl. oz)				
103. Soft drinks, diet (1 can = 1½ cup)	1½ cup (11 fl. oz)				
104. Turkish coffee (1 small cup = ¼ cup)	¼ cup (2 fl oz)				
105. Coffee/Nescafe or tea	1 cup				
106. Hot chocolate or cocoa	1 cup				
107. Beer, regular (1 can = 1½ cup)	1½ cup				
108. Wine: red, white, or blush	½ cup (4 fl. oz)				
109. Liquor: whiskey, vodka, gin, rum	1/6 cup (1.5 fl oz.)				
**Miscellaneous**					
110. Manaeesh, zaatar	1 large				
111. Manaeesh cheese	1 large				
112. French fries (12-15)	1 cup				
113. Chips: potato, corn, tortilla (9-13)	1 cup				
114. Falafel sandwich, medium	1 item				
115. Chawarma sandwich, medium	1 item				
116. Burgers Beef	1 item				

117.Burgers chicken	1 item				
118.Burgers fish	1 item				
119.Pizza	1 slice				

Are there any other foods not mentioned above that you usually eat at least once per week?

Other foods that you usually eat at least once /week	Usual serving size	Servings/week
