# Supplementary material for: Assessing Kidney Injury Biomarkers and OTA Exposure in Urine of Lebanese Adolescents Amid Economic Crisis and Evolving Dietary Patterns
Source: Toxins (Basel). 2025 Nov 30;17(12):577. doi: 10.3390/toxins17120577 (PMC12737349; doi:10.3390/toxins17120577)
Supplement: Supplementary file 1 [file toxins-17-00577-s001.zip › Sample.pdf]

Sample Number	KIM-1 (pg/ml)	NGAL (pg/ml)	NAG (mU/L)	OTα (µg/l)	OtA (µg/l)	Creatinine in Urine (mg/dL)	TProtein in urine (mg/dL)
1	900.3	151.2	84.5	0	0	220.470	16.500
2	353.4	95.5	104.6	0	0	52.8	160.2
3	1300.8	93.4	89.7	0	0	334.13	16.7
4	314.5	102.7	21.8	0	0	25.1	3.1
5	761.3	118.2	104.6	0.62	0	225.77	248.1
6	90.3	71.8	31.5	0	0	114.02	5.5
7	449.2	144	100.1	0.78	0	127.92	10
8	601.8	118.4	84.5	0	0	176.78	7.3
9	563.4	138.7	87.5	0	0	173.52	16
10	643.9	105.9	130	3.12	0	139.25	5.1
11	480.8	87.9	206.1	0	0	280.14	128.2
12	226.6	108.5	40.4	0	0	69.81	5.3
13	476.6	78.3	48.7	3.77	22.41	70.98	6.1
14	116.6	114.1	25.5	0.52	0	38.75	4.3
15	217.6	84.6	86.7	1.44	0	54.38	4.7
16	151.3	86.1	128.5	14.62	0	217.11	11.5
17	598.7	76.8	101.6	0	0	122.89	8.7
18	199.7	126.6	94.2	2.94	2.26	148.32	6.2
19	1437.1	101.2	148.7	0.28	0	233.76	31.5
20	303.9	64.2	43.4	0	0	50.81	3.6
21	162.4	108.7	19.6	0	0	21.81	3.1

22	310.8	66.1	37.5	0	0	48.54	3.6
23	296.6	85.3	94.2	0	0	64.28	7.7
24	265	97.7	60.6	0	0	89.17	4
25	498.2	92.4	194.2	0	0	157.9	105.9
26	496.1	60.8	101.6	1.39	0	207.77	12
27	81.8	81.7	109.1	0	0	82.7	3.8
28	203.9	108.5	74	0	0	70.46	11.4
29	447.1	72.1	42.7	0	0	122.4	6
30	118.2	76.3	68.8	1.45	0	28.98	3.3
31	516.6	35.9	62.1	1.18	0	89.47	3.7
32	277.6	97.2	78.5	3.96	0	148.07	10.1
33	780.8	76.9	119.6	2.87	0	198.69	8.5
34	396.6	111.5	82.2	1.3	0	75.57	7
35	532.9	77.3	115.1	2.38	0	145.02	5.1
36	206.1	61.6	56.9	0	0	71.7	3.3
37	306.6	99.5	86	0	0	101.06	5.6
38	249.2	62.4	54.6	0	0	57.17	4.4
39	275.5	64.8	86	0	0	44.38	5.3
40	584.5	93.5	149.4	12.18	0	204.52	79.5
41	251.3	75.3	62.1	0	0	90.27	6.3
42	390.3	69.1	130	0	0	158.91	11.1
43	1502.9	98.6	206.9	0	0	203.74	19.5
44	142.4	80.3	29.3	0	0	17.42	2.8
45	323.9	84.3	86	4.55	0	84.17	3.9
46	350.8	70.4	58.4	0	0	278.4	8.6
47	879.2	104.3	105.4	0.86	0	80.41	7.6

48	109.7	68.3	59.9	0	0	109.1	8.7
49	1009.7	125.4	197.2	0	0	238.02	10.7
50	652.4	118.3	338.2	2.06	0	101.25	6.8
51	400.8	73.7	215.1	1.28	0	144.8	10.9
52	550.8	79.9	206.9	0	0	57.78	4.5
53	317.1	48.7	239	0.46	0	47.22	4.2
54	349.7	101.2	68.8	0	0	60.5	4.4
55	257.6	39.6	74.8	2.66	0	98.15	5.9
56	838.2	109.3	99.4	4.51	0	115.73	6.5
57	639.2	157.9	104.6	11.71	0	76.33	4
58	328.7	141.6	135.2	4.08	0	67.65	4.1
59	535	112.7	146.4	8.02	0	105.9	5.2
60	398.7	62	71	3.8	0	42.1	4.6
61	568.2	70.2	186	9.3	0	125.02	8.4
62	313.4	43.7	71	0	0	33.02	4.6
63	1218.2	95	767.3	10.62	0	206.34	9.9
64	2026.1	68	224	17	0	334.03	12.7
65	348.7	57	95.7	8.44	0	125.78	9.7
66	309.2	137.6	98.7	0	0	107.32	7
67	1375	92	291.2	2.68	0	465.77	34
68	1692.4	88.8	291.2	0	0	417.63	26.5
69	661.3	86.8	118.8	0.42	0	179.89	25
70	246.6	89.1	127	0	0	112.37	7.9
71	301.3	22	202.4	3.19	0	90.55	6.5
72	292.9	27	156.9	0	0	198.19	13.2
73	1109.2	111.3	104.6	2.49	0	128.87	6.4

74	333.9	51.8	119.6	0	0	73.22	7.5
75	577.1	89.7	148.7	2.4	0	54.27	7.5
76	455.5	56.5	153.9	0	0	107.91	11.7
77	1213.4	73.3	134.5	3.46	0	162.44	13.9
78	857.6	102.3	137.5	0	0	205.32	6.4
79	624.5	84.4	182.2	2.95	0	299.59	11.7
80	128.7	80.5	150.1	0	0	99.59	6.8
81	112.4	57	26.3	0	0	32.13	2.4
82	403.4	89	137.5	0	0	125.15	4.4
83	327.6	61	93.4	2.15	0	105.46	6
84	415.5	72	221.8	0.87	0	93.4	8.4
85	397.1	77	86.7	0	0	100.53	4.2
86	753.4	82	170.3	0	0	399.96	13.2
87	201.3	59	80.7	0	0	49.85	5
88	777.1	67	106.9	1.04	0	129.98	5.9
89	462	47.3	1721.8	23.74	0	292.76	17.6
90	662	72.2	2368.8	40.52	0	146.68	5.8
91	582	63.3	94.9	11.89	0	72.46	5.2
92	252	74.9	70.3	0	0	162.28	5.1
93	552	68	47.2	1.04	0	189.68	65.1
94	292	59.6	56.1	2.84	0	162.02	7.1
95	2	49.3	195.7	0	0	29.48	2.8
96	202	49.2	145.7	6.16	0	194.01	7.5
97	822	38.6	82.2	4.67	0	123.13	10.2
98	552	76.7	86	3.71	0	239.63	18.8
99	712	42.1	28.5	0	0	37.87	4.7

100	222	72.5	93.4	0	0	38.88	3
101	492	78	38.2	0	0	32.89	2.7
102	332	78.4	31.5	0	0	142.65	6.1
103	52	70.8	59.9	3.58	0	145.35	5.7
104	22	33.7	65.8	0	0	142.35	4.4
105	552	61.9	47.2	0	0	222.4	14.5
106	382	72.8	28.5	10.48	0	189.38	13.5
107	682	48.6	45.7	0.02	0	11.86	2.3
108	322	47.2	79.3	0	0	223.07	19.9
109	432	51.5	21.8	2.65	0	67.75	3.3
110	402	56.6	69.6	5.98	0	277.55	41.9
111	112	73.4	69.6	11.76	3.04	155.02	21.9
112	272	72.1	241.2	9.49	0	209.35	7.7
113	872	71.7	124.8	0	0	338.34	9.5
114	892	77.6	113.6	0	0	206.71	14.3
115	722	47.9	127	3.06	19.98	143.81	17.6
116	262	46.3	100.9	0.87	0	34.69	3.3
117	562	73.7	168.1	0	0	27.79	2.1
118	272	76.7	52.4	0	0	109.82	9.8
119	102	72	94.2	0	0	173.95	15.8
120	2	51.4	75.5	5.18	1.09	124.4	11.6
121	662	33.1	130.7	0	0	84.16	6.1
122	692	45.2	142.7	0	0	113.31	10.1
123	832	58.1	65.1	0	0	162.17	3.6
124	312	63.8	89.7	1.27	0	83.56	5.9
125	522	42.4	146.4	0	0	131.56	5.3

126	552	34	84.5	0	0	102.04	6.4
127	202	41.4	77	1.08	0	64	23.2
128	412	38.8	53.9	0	0	87.58	13
129	332	52.8	84.5	1.73	0	69.11	14.5
130	642	39	177	5.28	0	82.25	4.4
131	542	42	43.4	0	0	51.09	3.4
132	152	68.1	56.9	0	0	31.77	2.5
133	402	34.7	52.4	3.66	0	194.91	6.4
134	422	42.2	49.4	6.85	0	162.5	5.7
135	62	48.3	63.6	0	0	46.08	3.3
136	0	44.1	40.4	0	0	74.7	4.2
137	392	67.2	21.8	1.53	0	65.54	5.3
138	812	77	52.4	0	0	72.67	4.1
139	742	61.2	59.1	0	0	121.93	7.2
140	512	60.9	50.9	2.68	0	138.07	12.5
141	672	33.3	109.1	1.67	0	41.26	2.4
142	562	32.1	177	0	0	64.15	7
143	0	48.5	52.4	3.22	0	145.02	6.2
144	112	42.9	62.8	0	0	95.74	9.1
145	112	43.7	70.3	1.83	0	167.86	17.8
146	822	59	95.7	5.28	0	70.52	6.2
147	622	42.8	109.1	0.31	2.02	124.4	11.6
148	422	41.1	54.6	0	0	181.83	12
149	502	45.4	58.4	0.63	0	241.5	16.5
150	502	44.8	115.1	0	0	49.42	8.5
151	0	40.7	38.2	0	0	172.19	5.6

152	0	62.4	33	41.14	0	159.84	7.3
153	0	66	52.4	4.97	0	146.01	13.9
154	752	33.8	83	0	0	47.08	6.3
155	852	49.3	97.2	6.94	0	302.15	37
156	612	40.2	35.2	2.48	0	164.8	7.4
157	502	41.8	262.1	0	0	63.46	4.9
158	382	54	86.7	0	0	48.26	3.5
159	0	48.2	69.6	0	0	121.7	18.9
160	112	38.8	21.8	0	0	45.2	7.8
161	52	76	114.3	0	0	31.9	2.8
162	612	31.2	99.4	0	0	96.32	6.7
163	522	61.9	26.3	0	0	16.13	2.4
164	72	51.7	41.9	2.06	0	173	20.2
165	382	49.7	25.5	0	0	126.2	7.1
166	0	52.2	144.2	2.28	2.38	58.11	5.2
167	0	52.7	91.2	11.82	3.38	213.49	14.9
168	412	55.7	66.6	1.97	0	132.5	8.4
169	52	49.6	103.1	2.38	0	162.17	3.6
170	802	45.8	93.4	1.01	3.23	237.54	6.7
171	642	57.6	37.5	0	0	55.05	6.7
172	242	57.8	89.7	0	0	87.36	3.7
173	422	67.5	120.3	0	0	174.74	7.9
174	332	57.2	59.1	0	0	217.1	15.7
175	62	39.8	43.4	0	0	104.52	10.1
176	302	38.9	166.6	2.76	0	111.2	10.5
177	534.7	61.3	2274	0	0	210.63	16.4

178	601.3	63.5	2665.8	0	0	171.84	19.5
179	244.7	30.5	175.5	0	0	61.37	4.5
180	141.3	68.8	168.1	0.54	0	140	5.6
181	198	59.7	68.8	1.72	2.1	112.58	10.6
182	204.7	73.8	198.7	83.6	0	94.33	5.5
183	91.3	71.6	119.6	0	0	254.17	14.5
184	168	58.7	85.2	0	0	44.99	3.8
185	381.3	47.5	119.6	0	0	8.73	2.5
186	474.7	65.8	60.6	0	0	36.88	4.7
187	301.3	73.7	30.7	0	0	118.86	18.8
188	131.3	62.5	41.9	0	0	146.11	6.5
189	178	72.3	103.9	0	0	28.81	17.1
190	194.7	54.6	83.7	0	0	26.33	3.7
191	0	69.3	64.3	0	0	12.8	2.3
192	151.3	47.3	49.4	2.57	5.3	103.4	15.5
193	248	82.8	29.3	0	0	24.02	10.1
194	414.7	70.7	110.6	2.43	0	98.86	46.5
195	208	65.9	55.4	2.87	0	207.05	14.7
196	214.7	55.4	380	6.81	23.57	154.98	10
197	214.7	70.8	105.4	3.3	0	113.27	4.5
198	234.7	69.4	137.5	2.27	0	62	6.2
199	54.7	43	91.2	0.6	0	138.55	10.3
200	158	78.4	94.9	0	0	14.83	2.2
201	301.3	39.6	124.8	0	0.06	44.32	4
202	341.3	32.6	35.2	0	0	23.28	5.1
203	308	56.2	63.6	5.08	3.24	246.97	20.5

204	174.7	64.8	44.2	3.43	0	81.7	14.3
205	234.7	68.9	155.4	0	0	33.78	2
206	198	46.7	256.1	1.55	0.08	194.27	28.4
207	0	51.3	45.7	1.96	0	37.35	2.5
208	184.7	31.8	152.4	0	0	137.33	8.7
209	168	29	41.2	12.05	0	25.04	3.2
210	321.3	40.2	124	143.12	0	33.75	4
211	278	51.8	50.1	5.4	0.65	301.12	26.1
212	114.7	40.7	51.6	1.46	0	95.17	4.4
213	108	50.5	207.6	0.1	0	42.84	7.5
214	101.3	46.4	57.6	28.46	0	112.26	10.7
215	0	43.4	41.9	24.03	1.34	111.02	11.7
216	91.3	61.1	128.5	239.51	0	109.22	7.4
217	114.7	58.2	305.4	0	0	44.33	8.4
218	308	47.2	290.4	0	0	24.1	2.9
219	141.3	38.7	103.1	0.46	0	148.16	9.6
220	148	39.1	46.4	5.47	0	93.62	12.4
221	84.7	60.6	71	6.11	0	95.91	8.1
222	91.3	35.9	237.5	0.65	0	37.7	3.7
223	0	31.4	108.4	0.25	0	17.52	2.4
224	158	49.1	48.7	2.2	0	229.81	8.7
225	161.3	34.3	106.1	4.83	0.84	221.94	4.2
226	318	40.5	127.8	1.9	0	181.22	16.4
227	168	39.1	81.5	1.4	0.19	62.39	5.6
228	141.3	35.9	22350.9	0.86	0	135.62	22.9
229	51.3	29.5	58.4	21.24	0	68.77	4

230	88	41.8	163.6	0	0	124.4	11.6
231	8	35.2	58.4	0.23	0	156.08	11.5
232	41.3	47.6	53.1	1.06	0	80.08	5.9
233	64.7	44.6	84.5	3.74	0	148.85	18.7
234	271.3	59.2	78.5	0	0	66.63	8.3
235	198	60.9	107.6	2.83	0	169.03	43.2
236	144.7	52.3	135.2	0.81	0	63.74	8.3
237	94.7	35.5	56.9	5.44	7.12	173.47	11
238	111.3	35.7	96.4	0	0	43.57	4.8
239	0	57.6	71	1.94	5.62	271.61	25.7
240	0	51.5	53.1	8.01	0	86.54	6.6
241	68	43.1	268.8	0.02	0	128.23	8.3
242	294.7	57.5	108.4	1	0	145.84	4.2
243	218	52.5	77	1.63	10.34	137.95	10.4
244	174.7	41.2	43.4	0.02	0	87.96	11.2
245	114.7	57.1	76.3	0	0	164.12	18.4
246	231.3	44.9	102.4	0	0	161.56	7.4
247	0	36.1	185.2	1.23	0	79.36	5.1
248	0	41.7	113.6	0	0	96.33	9.2
249	314.7	48.3	55.4	0	0	88.41	3.5
250	424.7	54.2	125.5	0.02	0	127.69	31.4
251	354.7	35.5	122.5	9.56	0	190.57	6.2
252	144.7	63.1	156.9	0	0	28.97	3.4
253	134.7	26.5	147.2	0.02	0	74.43	4.8
254	141.3	42.8	58.4	0	0	38.13	2.6
255	0	44.7	81.5	0.59	0	116.46	13.9

256	0	67.7	41.2	1.79	0	86.61	4
257	274.7	31.5	69.6	3.17	56.36	221.1	12.7
258	471.3	28.1	86	0	0.79	12.65	10.6
259	238	47.4	136.7	0	0	21.15	2.2
260	218	36.1	133	0.02	2.66	68.07	8.2
261	178	42	44.2	3.17	0	43.54	2.3
262	161.3	56.9	107.6	1.13	0	58.87	3.2
263	0	60.4	95.7	0	0	34.1	2.7
264	51.3	45.4	175.5	0.25	0	33.48	3.2
265	46.7	47.5	31.5	0	0	12.27	2.1
266	485	39.5	230.7	0	0	132.9	9.3
267	0	36.3	36.7	3.83	0	217.1	15.7
268	447.5	29.9	84.5	0	0	58.84	6.7
269	1034.2	51.7	56.9	1.49	0	73.63	3.4
270	0	37.2	79.3	0	0	111.2	10.5
271	71.7	72.9	66.6	2.41	0	85.49	6.2
272	460	38.9	26.3	4.09	0	78.26	4.6
273	339.2	30.4	83	0.32	0	103.3	8.5
274	1032.5	58.1	653.1	0	0	415.16	121.9
275	125.8	48.2	39.7	0.21	0	22.98	2.4
276	735.8	30.7	150.1	0	0	256.84	28
277	0	27.9	36.7	0	0	36.73	3.7
278	785.8	61.9	47.9	18.58	0	44.98	3.8
279	63.3	62.8	59.1	0	0	115.22	9.5
280	0	49.5	59.1	0	0	178.37	13.4
281	51.7	32.8	113.6	0.77	0	201.6	15.9

282	500	36.5	209.9	1.44	0	158	20.6
283	0	36.1	83	0	0	126.53	10
284	635	52.6	44.2	0.45	0	35.86	2.9
285	476.7	42.9	134.5	3.19	0	108.52	11.1
286	45.8	36.4	56.9	0.1	0	62.11	4.6
287	100	57.5	51.6	0	0	65.2	7.2
288	0	43.8	42.7	3.87	0.1	118.65	7.9
289	0	32.7	48.7	1.31	0	36.12	3.2
290	25.8	40.8	65.8	6.01	0	104.62	11.8
291	13.3	29.6	61.3	0	0	31.81	2.2
292	0	30.6	61.3	0.02	0	94.09	8.7
293	40	34.9	100.1	1.48	0	68.77	4
294	0	28.4	26.3	0	0	10.34	3
295	0	37.9	36	0.78	0	59.29	5
296	389.2	51.6	159.9	12.69	1.67	309.38	21.6
297	0	28.6	63.6	0.23	0	112.72	13.4
298	451.7	50.1	31.5	0	0	45.63	6.5
299	255.8	66.3	114.3	11.29	0.75	271.87	12.4
300	181.7	69.2	44.2	0.49	0	46.15	4
301	29.2	28.8	65.1	3.61	0	114.84	12
302	0	42.8	158.4	1.28	0	196.54	17.9
303	0	32	85.2	0.02	0	88.35	4.3
304	598.3	45	73.3	3.85	0	170.49	20.3
305	13.3	50	147.9	5.16	0	74.76	5.5
306	0	36.1	136.7	0	0	137.08	13.6
307	0	58.4	50.9	4.63	0	28.2	2.7

308	0	35.9	171	0	0	194.54	23.9
309	0	30.2	82.2	0	0	78.89	8.8
310	0	40.2	89.7	4.24	0	233.43	25.5
311	0	57.5	39	0	0	209.88	11.4
312	0	62.7	124.8	8.6	0	231.56	29.1
313	85.8	59	106.1	0	0	123.86	8.8
314	126.7	54.8	59.1	0	0	84.86	4.9
315	0	54.7	83	13.98	17.24	93.56	5.3
316	917.5	55.2	108.4	14.69	0	238.82	12.9
317	0	41.5	97.9	0	0	172.89	18.3
318	0	56.4	100.1	0	0	93.17	11.9
319	0	45.6	33	0.3	0	91.66	8.1
320	34.2	50.2	100.9	0.37	0	76.03	15.8
321	0	35.6	50.9	0.25	0	43.7	2.4
322	460.8	52.2	151.6	6.2	0	229.13	10
323	0	34.9	60.6	0	1.1	31.51	14.4
324	0	46.5	41.2	2.33	0	69.35	3
325	0	31.1	45.7	0.58	0	71.48	5.4
326	0	34.5	43.4	1.55	1.98	64.87	6.2
327	0	33.4	41.9	2.12	0	92.71	8.1
328	0	34.6	24	0.79	0	140.86	16.3
329	0	39.5	124.8	0	0	151.02	22.1
330	3.3	28.7	29.3	0	0	32.12	3.7
331	474.2	45.9	70.3	0	0	210.11	9.5
332	34.2	43.4	96.4	1.93	0	225.2	18.6
333	635	63.4	53.9	0	0	26.71	2.3

334	0	56.3	48.7	0	0	96.63	5.9
335	274.2	54.5	97.9	1.09	0	168.31	19.3
336	172.5	49.8	33.7	0	0	48.32	4.9
337	75	41.9	53.9	0.96	0	140.67	3.8
338	67.5	68.2	83.7	0.29	0	180.18	5.5
339	0	54.9	41.9	0.27	0	121.62	3.5
340	0	66.8	79.3	6.74	3.45	77.1	5.7
341	0	60.3	114.3	0.1	0	47.61	5.3
342	94.2	31.3	115.8	0.35	0	58.71	8.5
343	23.3	46.1	86.7	2.27	1.27	309.71	25.6
344	194.2	54.6	24.8	0	0	60.72	3
345	90.8	31.7	90.4	0	0	86.93	8.9
346	260	31.7	179.3	2.36	1.82	243.28	11.6
347	60	46	59.9	1.89	0	123.44	5.1
348	111.7	47	78.5	54.92	65.92	301.41	13
349	65.8	32.9	83.7	1.3	0	87.64	3.5
350	0	45.4	33	1.44	0	61.53	4.9
351	25	35.6	112.8	1.02	0	136.05	9.7
352	105	37.5	36	2.64	0	105.6	3.8
353	185.5	85.6	55.4	0	0	86.11	3.3
354	286.8	60.4	124	15.71	23.2	112.3	9.2
355	395.9	52.2	116.6	0	0	178.68	6.3
356	479.5	46.9	74	4.74	0	118.42	6.3
357	569.1	49.5	42.7	1.42	0	250.81	8.2
358	347.7	34.1	114.3	0.25	0.62	87.78	12.1
359	214.5	31.7	22.5	0.36	0	67.27	2.8

360	64.1	48.8	3.9	0	0	35.18	2.2
361	125.5	43.1	86.7	0.88	3.47	111.55	10.8
362	429.5	46.3	53.1	1.37	0.95	125.44	3.9
363	399.1	33.2	74	1.51	0	104.33	13.2
364	241.8	28.6	50.1	0	0	41.12	3.4
365	662.3	67.2	56.1	4.44	8.96	165.64	13.2
366	1313.2	66.2	207.6	2.45	3.74	242.14	25.6
367	595.9	51.3	307.6	6.23	21.99	404.27	66.4
368	356.8	32.6	90.4	2.98	8.8	131.03	13.6
369	118.6	53.1	159.9	1.09	5.19	151.43	19.7
370	168.2	77.4	58.4	0.26	0	76.94	4.2
371	235	27.9	56.1	0.91	1.12	217.28	7.3
372	569.1	28.8	33	0.43	0	20.12	19.4
373	612.3	32.9	40.4	0	0.95	109.57	4.9
374	413.6	27.3	40.4	0	0	47.11	4.1
375	316.4	34.8	62.1	0.63	1.83	403.04	7.5
376	170.5	27.4	9.9	0.55	0	64.41	6.1
377	319.1	57.4	80	0.26	0.81	196.97	8.2
378	243.6	38.1	112.8	1.44	0	124.4	11.6
379	376.4	44.8	150.1	4.18	0	166.66	15.7
380	625	51.3	81.5	2	5.07	275.51	10.2
381	365	33	71	2.31	2.34	117.01	1.7
382	476.8	72.2	151.6	4.33	2.04	267.29	15.7
383	264.5	46.5	98.7	6.85	0	480.09	15.4
384	50	33	21.8	0.77	1.31	115.23	7.7
385	170.9	44.8	50.9	0.67	0	151.05	10.2

386	470.9	60.3	143.4	4.28	16.08	217.79	6.6
387	532.3	56.9	73.3	0.94	2.32	80.89	10.1
388	421.4	50.7	130	2.24	0	161.36	5.6
389	371.4	37.5	55.4	0.38	0	93.17	4.1
390	594.1	63.1	138.2	0.77	0	151.78	7.7
391	323.6	32.9	45.7	1.33	0	114.72	7.9
392	310.9	53.9	63.6	0	0.74	65.1	4.9
393	1138.6	54.4	95.7	2.81	5.33	278.99	13.7
394	595.9	29.2	147.2	2.04	0	111.89	9.5
395	900.5	75.5	127	1.97	12.54	182.22	24.1
396	470.5	43	60.6	2.44	0	109.59	9.9
397	816.8	40.7	95.7	0.63	0	290.07	13.1
398	538.2	64.5	113.6	0.21	0	129.8	6.2
399	368.6	49	44.2	0	0	107.58	4.2
400	495.5	32.5	150.1	0.95	0	106.08	13
